# Improved folate accumulation in genetically modified maize and wheat

**DOI:** 10.1093/jxb/ery453

**Published:** 2019-02-08

**Authors:** Qiuju Liang, Ke Wang, Xiaoning Liu, Bisma Riaz, Ling Jiang, Xing Wan, Xingguo Ye, Chunyi Zhang

**Affiliations:** 1 Biotechnology Research Institute, Chinese Academy of Agricultural Sciences, Beijing, China; 2 Institute of Crop Science, Chinese Academy of Agricultural Sciences, Beijing, China

**Keywords:** Aminodeoxychorismate synthase, biofortification, folates, GTP cyclohydrolase I, maize, wheat

## Abstract

Folates are indispensable co-factors for one-carbon metabolism in all organisms. In humans, suboptimal folate intake results in serious disorders. One promising strategy for improving human folate status is to enhance folate levels in food crops by metabolic engineering. In this study, we cloned two *GmGCHI* (GTP cyclohydrolase I) genes (*Gm8gGCHI* and *Gm3gGCHI*) and one *GmADCS* (aminodeoxychorismate synthase) gene from soybean, which are responsible for synthesizing the folate precursors pterin and p-aminobenzoate, respectively. We initially confirmed their functions in transgenic Arabidopsis plants and found that *Gm8gGCHI* increased pterin and folate production more than *Gm3gGCHI* did. We then co-expressed *Gm8gGCHI* and *GmADCS* driven by endosperm-specific promoters in maize and wheat, two major staple crops, to boost their folate metabolic flux. A 4.2-fold and 2.3-fold increase in folate levels were observed in transgenic maize and wheat grains, respectively. To optimize wheat folate enhancement, codon-optimized *Gm8gGCHI* and tomato *LeADCS* genes under the control of a wheat endosperm-specific glutenin promoter (*1Dx5*) were co-transformed. This yielded a 5.6-fold increase in folate in transgenic wheat grains (*Gm8gGCHI*^+^/*LeADCS*^+^). This two-gene co-expression strategy therefore has the potential to greatly enhance folate levels in maize and wheat, thus improving their nutritional value.

## Teaser

A two-gene co-expression strategy to increase the folate precursors pterin and p-aminobenzoate increases the levels of folates in wheat and maize grains.

## Introduction

Tetrahydrofolate and its derivatives are collectively termed folates and they belong to a group of water-soluble vitamin B compounds (B9) (Hanson and Gregory, 2002; Basset *et al.*, 2005). As essential carriers and donors of one-carbon units, folates are involved in multiple metabolic processes, including biosynthesis of purine, thymidylate, methionine, serine, pantothenate, and formylmethionyl-transfer RNA, providing methyl groups for most cellular methylation reactions (Scott *et al.*, 2000). Folates play roles in histidine degradation pathways in mammals, including humans (Hilton *et al.*, 2003). In addition, folates are necessary for biosynthesis of lignin, alkaloids, and chlorophyll (Hanson and Gregory, 2011). The significant roles played by folates indicate that they are indispensable micronutrients for all living organisms.

Microbes and plants can synthesize folates *de novo*, but mammals (including humans) lack the complete biosynthesis system and hence are unable to produce folates. Therefore, they must rely on dietary sources, mainly from plants. Folate levels vary among food sources. Eggs, liver, green leafy vegetables, and leguminous vegetables are high in folates, whereas some staple foods such as rice, wheat, and potatoes contain little (Bekaert *et al.*, 2008; Blancquaert *et al.*, 2010). Folate malnutrition is a global health problem that occurs even in developed countries. Folate deficiency increases the risk of many diseases, including infant neural tube defects, megaloblastic anemia, cardiovascular disease, and certain cancers (Li *et al.*, 2003; Fenech, 2010; Nazki *et al.*, 2014; Herrera-Araujo, 2016). Currently, fortified foods or synthetic folic acid pills are used to alleviate folate deficiency in Western countries (Blancquaert *et al.*, 2014), but it would be difficult to implement such dietary intervention across all populations. Moreover, excessive uptake of synthetic folic acid has caused much concern. As our knowledge of folate biosynthesis and metabolism in plants has improved, engineering solutions for biofortification of food crops by enhancing their natural folate content have attracted much attention (Blancquaert *et al.*, 2014; Strobbe and Van Der Straeten, 2017).

**Fig. 1.**
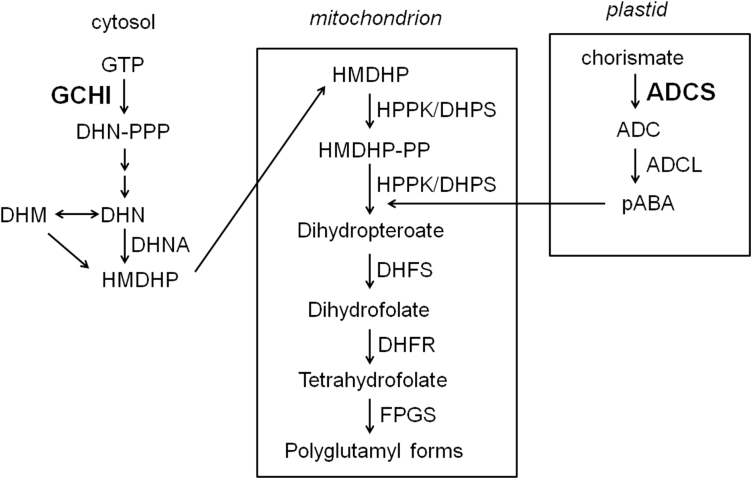
Outline of folate biosynthesis pathways and related catalysing enzymes in plants. Pterins and para-aminobenzoate (pABA) are synthesized in the cytosol and plastids, respectively. The two precursors are coupled and glutamylated in the mitochondrion. The engineered enzymes GCHI (GTP cyclohydrolase I) and ADCS (aminodeoxychorismate synthase) are marked in bold. DHNA, DHN aldolase; ADCL, aminodeoxychorismate lyase; HPPK/DHPS; 6-hydroxymethyldihydropterin pyrophosphokinase/dihydropteroate synthase; DHFS, dihydrofolate synthase; DHFR, dihydrofolate reductase; FPGS, folylpolyglutamate synthase; DHN, dihydroneopterin; DHM, dihydromonapterin; HMDHP, 6-hydroxymethyldihydropterin; -PP, pyrophosphate; -PPP, triphosphate.

The folate biosynthesis pathways of Arabidopsis have been extensively investigated. At least 10 enzymes take part in this process, most of which have been cloned and functionally confirmed by *in vitro* assays or by complementation analysis (Ravanel *et al.*, 2001; Basset *et al.*, 2002, 2004; Sahr *et al.*, 2006). Tetrahydrofolate (THF) is a tripartite molecule composed of pterin, p-aminobenzoate (pABA), and glutamate moieties. In plants, folate biosynthesis is highly compartmented; pterin and pABA are synthesized in the cytosol and plastids, respectively, and subsequent coupling of these two precursors and the addition of glutamate moieties takes place in mitochondria (Fig. 1) (Basset *et al.*, 2005). GTP cyclohydrolase I (GCHI) and aminodeoxychorismate synthase (ADCS) act as the initial enzymes in the formation of pterin and pABA, respectively (Basset *et al.*, 2002, 2004). It has been proposed that the reactions catalysed by these two enzymes act as rate-determining steps (Hossain *et al.*, 2004).

The first attempt to increase folate content was by overexpression of *GCHI*. Overexpression of the *folE* gene encoding GCHI from *E. coli* in Arabidopsis yielded 1250-fold and ~2–4-fold increases in pterin and folate levels, respectively (Hossain *et al.*, 2004). By inducing overexpression of the mammalian *GCHI* gene under fruit-specific promoters, pterin and folate contents of tomato fruit were increased by ~3–140 times and two times, respectively (Díaz de la Garza *et al.*, 2004). This strategy was also applied to lettuce, corn, and common bean, resulting in 8.5-, 2-, and 3-fold increases in their folate contents, respectively (Naqvi *et al.*, 2009; Nunes *et al.*, 2009; Ramírez Rivera *et al.*, 2016). Large increases in pterin levels of transgenic plants confirmed the key role of *GCHI* in promoting folate biosynthesis (Díaz de la Garza *et al.*, 2004; Hossain *et al.*, 2004). The relatively low levels of folate accumulation in these transgenic plants were presumed to be due to depletion of pABA, the other folate precursor, and further increases in folate levels were achieved by boosting pABA provision (Díaz de la Garza *et al.*, 2004). A two-gene *GCHI* and *ADCS* simultaneous expression strategy proved very successful in tomato and rice, yielding 25-fold and 100-fold increases in folate levels, respectively (Díaz de la Garza *et al.*, 2007; Storozhenko *et al.*, 2007). Attempts to overexpress other folate biosynthesis genes in rice, or to couple them with *GCHI*, produced only slightly higher folate levels (Dong *et al.*, 2014). A study of folate-biofortified rice indicated that its stability in storage can be improved by using animal folate-binding proteins (Blancquaert *et al.*, 2015). A modest (3-fold) folate increase was observed in biofortified potatoes by co-expression of *GCHI* and *ADCS* (Blancquaert *et al.*, 2013), and a greater folate enhancement (9-fold) was obtained by the additional introduction of the other two folate biosynthesis genes *HPPK/DHPS* (6-hydroxymethyldihydropterin pyrophosphokinase/dihydropteroate synthase) and *FPGS* (folylpolyglutamate synthase) (De Lepeleire *et al.*, 2018). These studies indicated the feasibility of folate biofortification in plant foods.

Soybeans (*Glycine max*) accumulate large amounts of folates. In contrast, the grains of maize and wheat accumulate them poorly. Therefore, we are seeking to use the folate biosynthesis genes from soybeans to improve folate contents of maize and wheat; however, the genes involved in soybean have not yet been functionally characterized. In this study, we isolated the two key soybean folate biosynthesis genes, *GmGCHI* and *GmADCS*, and confirmed their function in Arabidopsis. Previously, a 2-fold increase of folate content has been observed in white corn after overexpression of *folE* from *E. coli* (Naqvi *et al.*, 2009). In this study we therefore enhanced the folate content of maize by achieving simultaneous expression of *GmGCHI* and *GmADCS* via endosperm-specific promoters. As folate biofortification in wheat has not been attempted previously, we co-expressed *GCHI* and *ADCS* from soybean and tomato to boost the folate metabolic flux. We observed significant enhancement of folate levels in the transgenic maize and wheat grains.

## Materials and methods

### Plant materials and growth conditions

The plant materials used in this study included the soybean (*Glycine max*) cultivar Zhonghuang24 provided by Prof. Wang Lei of the Biotechnology Research Institute of the Chinese Academy of Agricultural Sciences. We obtained the wheat (*Triticum aestivum*) cultivar Fielder from the National Crop Germplasm Bank at the Institute of Crop Science of the Chinese Academy of Agricultural Sciences. We obtained the *Arabidopsis thaliana* ecotype Columbia-0, the maize (*Zea mays*) cultivar line HiII and the maize inbred line Zheng58 from sources sustained in our laboratory.

The soybean plants were grown in a greenhouse at a temperature of 25 °C under 12/12 h light/dark conditions. We grew the Arabidopsis plants in an air-conditioned greenhouse maintained at a temperature of 22 °C. The light intensity was set to 300 μmol m^–2^ s^–1^ under a 16/8 h light/dark cycle. We transformed the plants *in planta* just before they reached the flowering stage. The maize plants were grown in a sunlit greenhouse at 25 °C under a 12/12 h light/dark cycle. Immature wild-type embryos aged 10–12 d post anthesis were used for *Agrobacterium-*mediated transformation. The wheat plants were grown in a greenhouse at 22 °C under a 16/8 h light/dark cycle. Immature wild-type embryos at post-anthesis ages of 14–15 d were used for *Agrobacterium-*mediated transformation.

### Expressed sequence tags used to obtain the target soybean genes

We BLASTed the Arabidopsis *AtGCHI* and *AtADCS* coding sequences against the soybean expressed sequence tag (EST) database on the NCBI website (https://blast.ncbi.nlm.nih.gov/Blast.cgi), and short and disconnected ESTs were found. There were four ESTs with high similarity to the *AtGCHI* 5´ terminal sequence and 11 ESTs similar to the 3´-terminal sequence. Two ESTs were similar to the 5´-terminal sequence of *AtADCS*, one EST was similar to the *AtADCS* middle sequence, and eight ESTs were similar to the 3´-terminal sequence (see Supplementary Fig. S1 at *JXB* online). We then used the resulting ESTs as a basis for assembling and amplifying the *GmGCHI*s and *GmADCS* coding sequences.

### RNA extraction and gene full-sequence cloning by RACE

To clone the target genes, we collected young soybean leaves from 20-d-old seedlings, extracted total RNA using a UGENE Total RNA KIT II (Unipro) according to the manufacturer’s instructions, and then used a First-Strand DNA Synthesis Kit (Toyobo) to synthesize single-stranded cDNAs. For cloning of *GmGCHI*s, first, two partial sequences (700 bp products, named M1, M2) were obtained using primers, namely *GTPCHI-EST1-FW + GTPCHI-EST8-RV* and *GTPCHI-EST3-FW + GTPCHI-EST6-RV* (Supplementary Figs S1B, S2A, primer sequences are shown in Supplementary Table S1). Subsequently, we found that sequences of 5´EST-1, M1, and 3´EST1/8 could overlap and be assembled into a 1200-bp sequence, and that 5´EST-3, M2, and 3´EST6 could overlap and be assembled into a 1180-bp sequence. After a BLAST search in the genomic database of *Glycine max*, the two sequences were found to be respectively located in chromosome 8 (8g46160) and chromosome 3 (3g21540), and were named as *Gm8gGCHI* and *Gm3gGCHI*. We used the 5´RACE and 3´RACE techniques to complete the *GmGCHI*s sequences. Total RNA was treated according to instructions provided by the manufacturer of the 3´-RACE System for Rapid Amplification of cDNA Ends and the 5´-RACE System for Rapid Amplification of cDNA Ends (Invitrogen). We designed the specific primers *Gm8gGCHI5´RACE*, *Gm8gGCHI5´RACE nest*, *Gm8gGCHI3´RACE*, *GmGCHI8g3´RACE nest*, *Gm3gGCHI5´RACE*, *Gm3gGCHI5´RACE nest*, *Gm3gGCHI3´RACE*, and *GmGCHI8g3´RACE nest* (Supplementary Fig. S1B, Table S1) and operated the PCR system following the manufacturer’s instructions (Fig. S2B, C). We obtained a 66-bp 5´ untranslated region (UTR), a 1380-bp coding sequence (CDS), and 177-bp 3´-UTR for *Gm3gGCHI*, and a 323-bp 5´-UTR, a 1374-bp CDS, and 784-bp 3´-UTR for *Gm8gGCHI*. Finally, we obtained full coding sequences of the following *GmGCHI*s using primers: *Gm3gGCHIF1*, *Gm3gGCHIR1*, *Gm8gGCHIF1*, and *Gm8gGCHIR1* (Supplementary Figs S1B, S2D). For amplification of *GmADCS*, a 1352-bp product was obtained with *mESTF1*+*3´EST5-R2* (Supplementary Figs S1B, S2E). Following a BLAST search in the *Glycine max* database, the 1352-bp product was located in chromosome 10 (10g35580), and the location predicted a 2304-bp coding sequence. Based on this, a 2331-bp product was obtained with the primers *ADCSMF* + *ADCSMR.* Next, the full cDNA sequence of GmADCS was obtained by RACE technology with the primers *ADCS5´RACE*, *ADCS5´RACE nest*, *ADCS3´RACE*, and *ADCS3´RACE nest* (Supplementary Figs S1B, S2F, G, Table S2) and it consisted of a 218-bp 5´-UTR, a 2784-bp CDS, and a 252-bp 3´-UTR. Finally, we obtained the full coding sequences of *GmADCS* with the primers *ADCS10FLF1* and *ADCS10R1* (Supplementary Table S2). We took the codon preferences of wheat as the basis for optimization of the full *Gm8gGCHI* and *LeADCS* coding regions, which were then synthesized directly by Jinsirui Biotech Co. (Nanjing).

### Conserved domain analysis and phylogenetic tree of GmGCHI and GmADCS

We used the Conserved Domain Database (CDD) on the NCBI website (https://www.ncbi.nlm.nih.gov/Structure/cdd/wrpsb.cgi) to predict the conserved domains of the GmGCHI and GmADCS protein sequences. We used the ClustalW2 service (https://www.ebi.ac.uk/Tools/msa/clustalw2/) to perform multiple alignments of GCHI homologs and ADCS homologs. The phylogenetic trees were constructed using the MEGA4.0 software by use of the Neighbor-Joining analysis of Bootstrap Test of Phylogeny, with the Bootstrap set as 500 replicates.

### Construction of GFP fusion proteins and subcellular localization analysis

We amplified full *GmGCHI* coding sequences with the terminating codon deleted and a 360-bp N terminal *GmADCS* sequence and cloned them in the correct direction into the *Bgl* II and *Kpn* II sites of a *pRTL2* vector, respectively. The target sequences were fused with the green fluorescent protein (GFP) coding regions. We isolated chloroplasts from Arabidopsis leaves grown in a greenhouse for 21 d and induced transient expression by applying the method described previously by Yoo *et al.* (2007). We captured the GFP signal using a laser-scanning confocal microscope (LSM700; Carl Zeiss).

### Construction and transformation of the overexpression vectors for Arabidopsis

We cloned the full *GmGCHI* and *GmADCS* coding sequences into the *pCAMBIA1302* binary vector construct at the *Nco* I/*Pml* I and *Bgl* II/*Pml* I sites, respectively, under the control of the CaMV35S promoter. These constructs were transformed into the Arabidopsis wild-type by the floral-dipping method (Zhang *et al.*, 2006). T_1_ grains were harvested and plated on half-strength Murashige–Skoog culture medium supplied with 25 mg l^–1^ hygromycin B, and the surviving green seedlings were transplanted into soil. Transgenic plants were identified by conducting a PCR analysis using the following gene-specific primers: *Gm3gGCHI-FSP*, *Gm3gGCHI-RSP*, *Gm8gGCHI-FSP*, *Gm8gGCHI-RSP*, *GmADCS-FSP*, and *GmADCS-RSP* (see Supplementary Fig. S1B, Tables S1, S2). Homozygous T_3_ transgenic plants were used for folate detection analysis. We obtained Arabidopsis plants containing both the *GmGCHI* and the *GmADCS* genes by crossing the *GmGCHI*-overexpressing plants with the *GmADCS*-overexpressing plants.

### Construction and transformation of overexpression vectors for maize and wheat

To support stable transformation of maize and wheat, we used a *pCAMBIA3301* vector containing the phosphinothricin (PPT) resistance gene as a basic vector skeleton. Digestion with the two blunt-end enzymes *Sma* I and *Pml* I and self-ligase resulted in deletion of the 35S promoter and *GUS* sequences from the *pCAMBIA3301* vector. The full *GmADCS* coding sequence was introduced into a *pHP20754* vector that contained the maize endosperm-specific promoter *Leg1A* and the *Leg1A* terminal sequences, at the *Nco* I and *Pml* I digestion sites. The full CDS sequences of the *Gm8gGCHI* gene were cloned into the *pHP20754* vector at the *Nco* I and *Pml* I sites, and then 2109 bp of the rice endosperm-specific promoter *GluC* was cloned into the vector at the *Eco*R V and *Nco* I sites to substitute the pristine maize *Leg1A* promoter. Next, the *Gm8gGCHI* expression cassette was cloned into the *GmADCS*-*pHP20754* vector at the *Sna*B I site. Finally, the complete *Gm8gGCHI* and *GmADCS* cassettes were introduced into the truncated *pCAMBIA3301* vector at the *Bst*E II site.

The complete *Gm8gGCHI-GmADCS-pCAMBIA3301* vector was transformed into *Agrobacterium tumefaciens EHA105* and introduced into maize cultivar HiII as described previously (Frame *et al.*, 2002). The same vector was transformed into *A. tumefaciens C58C1* and introduced into the wheat cultivar Fielder genome by methods described previously (Ishida *et al.*, 2015; Wang *et al.*, 2017).

Codon-optimized *Gm8gGCHI* and *LeADCS* genes were cloned onto a double T-DNAs vector *pWMB122* driven by a wheat endosperm-specific glutenin (*1Dx5*) promoter (Wang *et al.*, 2017) and transformed into *A. tumefaciens C58C1* and introduced into immature embryos of the wheat cultivar Fielder using methods described previously (Ishida *et al.*, 2015; Wang *et al.*, 2017).

### Identification of transgenic maize and wheat plants

Positive maize and wheat transgenic seedlings were analysed by PCR using the method described above for detecting transgenic Arabidopsis plants. We achieved stable inheritance by transferring the T-DNA expression cassette into a background genetic material of the maize inbred line Zheng58 by pollinating the transgenic maize HiII cultivar with Zheng58 pollen. We produced generations BC1~BC5 after backcrossing with Zheng58 five times. We confirmed successful production of stable transgenic maize lines by applying a previously described Southern blotting method (Wang *et al.*, 2017), then conducted RT-PCR analysis to detect the transcripts of the target genes in immature 25-d-old kernels of the transgenic plants. Total RNA was extracted with an RNA Extraction Kit supplied by Yuanpinghao Biotech Co. (Tianjin, China) and cDNA was synthesized using a RevertAid First Strand cDNA Synthesis Kit (Fermentas). The cDNA templates were subjected to PCR using the primer pairs *Gm8gGCHI-FSP* and *Gm8gGCHI-RSP*, and *GmADCS-FSP* and *GmADCS-RSP* (Supplementary Tables S1, S2) under the following conditions: 94 °C for 5 min, then 94 °C for 30 s, 58 °C for 30 s and 72 °C for 30 s for 32 cycles. We used wheat and maize *Actin* genes as internal controls and performed PCR amplification using the following primers: *TmActinF1*, *TmActinR1*, *ZmActinF1*, and *ZmActinR1* (Supplementary Table S2) and the same PCR conditions as above, but with only 28 cycles. The PCR products were analysed in a 1% agarose gel and photographed using a Peiqing gel system (Shanghai, China). We applied a haploid-doubling technique via anther culture (Yin *et al.*, 2018) to obtain homozygous transgenic wheat plants, then confirmed successful completion by fluorescence *in situ* hybridization according to methods described previously (Guo *et al.*, 2016).

We confirmed successful integration of the optimized *Gm8gGCHI* and *LeADCS* sequences into transgenic wheat plants by PCR amplification of their complete coding regions using the primers *Gm8GCHIPF1*, *Gm8GCHIPR1*, *LeADCSPF1*, and *LeADCSPR1*. We obtained the transcripts of target genes in the pollinated, immature, 25-d-old wheat kernels by RT-PCR using the primers *Gm8gGCHI-PFSP*, *Gm8gGCHI-PRSP*, *LeADCS-FSP*, *LeADCS-RSP*, and *TmActinF*, *TmActinR* (Supplementary Tables S1, S2) under the same PCR conditions as described above.

### Determination of levels of folate and its precursors contained in transgenic plants

We collected Arabidopsis leaves after 30 d growth in a greenhouse and mature maize and wheat grains after desiccating them for 48 h at 37 °C, and measured their folate, pterin, and pABA contents using a previously described method with some modifications (Lu *et al.*, 2007). Briefly, we used ~0.05 g of the tissues and ground them to a powder in liquid nitrogen. We immediately added 1000 μl of freshly prepared extraction buffer (5 mM phosphate buffer containing 0.5% sodium ascorbate and 0.1% 2-mercaptoethanol, pH 7.2) to the powder via a 1.5-ml tube before thawing the sample. After homogenizing the mixture, we immediately boiled it for 10 min, cooled it on ice for 10 min, and centrifuged it at 16 000 *g* at 4 °C for 10 min. We deconjugated the polyglutamylated tails by adding 35 μl of rat serum to the supernatant and incubating it at 37 °C for 4 h. The samples were then boiled again for 10 min, cooled on ice for 10 min, and centrifuged at 16 000 *g* at 4 °C for 10 min. The supernatant was subjected to 3-kDa ultra-filtration and then used for LC-MS/MS analysis.

Chromatographic analyses were performed on a 1260 HPLC system (Agilent) using an Akzo Nobel analytical column (Kromasil 100–5 C18, 50×2.1 mm) at a flow rate of 0.30 ml min^–1^. The injection volume was 15.0 μl. The temperature of the injector and column oven were separately maintained at 4 °C and 25 °C, respectively. The mobile phases were 0.1% (v/v) formic acid in water (phase A) and 0.1% (v/v) formic acid in acetonitrile (phase B). The gradient program was a total of 19 min. The proportion of mobile phase B was increased linearly from 5 to 9% over 2 min. In the following 6 min, phase B increased to 9.6% and was then sharply increased to 20% over 0.2 min. After holding at 20% for 3 min, the proportion of phase B was decreased to 5% in 0.2 min followed by a subsequent equilibration. An Agilent 6420 triple-quadruple tandem MS coupled to an ESI (electron spray ionization) interface was used for mass analyses and quantification of target analytes. The mass spectrometer was operated in positive ion mode. The parameters were optimised for the target analytes with a gas temperature of 350 °C, drying gas flow at 11 l min^–1^, nebuliser pressure at 35 psi, and capillary voltage at 3500 V (+). System operation, data acquisition and data analyses were performed using the MassHunter software. Hydroxymethylpterin (*m*/*z* 194–106, 30 eV), pterin-6-carboxylic acid (*m*/*z* 208–162, 20 eV), p-aminobenzoate (pABA, *m*/*z* 138–65, 30 eV), 5,10-methenyltetrahydrofolate (5,10-CH=THF, *m*/*z* 456–412, 30 eV), 5-methyltetrahydrofolate (5-M-THF, *m*/*z* 460–313, 20 eV), 5-formyltetrahydrofolate (5-F-THF, *m*/*z* 474–327, 20 eV), tetrahydrofolate (THF, *m*/*z* 446–299, 20 eV), and methotrexate (MTX, *m*/*z* 455–308, 30 eV) purchased from Schirck Laboratories (Jona, Switzerland) were used as standards. We repeated each sample preparation five times.

### Statistical analysis

Five biological replicates were used for detection analysis of each sample and results are shown as the means of all biological replicates. Statistical analysis of data was performed using *t*-tests in Microsoft Excel 2010.

### Accession numbers

Tomato GCHI (NP_001234141), *Arabidopsis thaliana* GCHI (AT3G07270), *Homo sapiens* GCHI (NP_000152), *E. coli* folE (NP_288736), tomato ADCS (NP_001234467), *Arabidopsis thaliana* ADCS (AT2G28880), and *E. coli* pabB (AGEC1).

## Results

### Cloning of the *GmGCHI* and *GmADCS* genes from soybean

Leguminous plants such as soybean are good sources of folate. Therefore, it is important to be able to clone the coding regions of *GCHI* and *ADCS* from soybean for genetic modification of cereal plants. By using the Arabidopsis *AtGCHI* and *AtADCS* coding sequences as query sequences, we found several short and disconnected ESTs related to soybean *GCHI* and *ADCS* (Supplementary Fig. S1). Partial coding sequences of two *GmGCHI* genes and one *GmADCS* gene were assembled and amplified using specifically designed primers (Supplementary Tables S1, S2, Fig. S2). We then applied a RACE technique to obtain the full coding sequences of *GmGCHI* and *GmADCS*. The two *GmGCHI* genes were located on chromosome 3 (3g21540) and chromosome 8 (8g46160), and were named *Gm3gGCHI* and *Gm8gGCHI*, respectively, and *GmADCS* was located on chromosome 10 (10g35580).

**Fig. 2.**
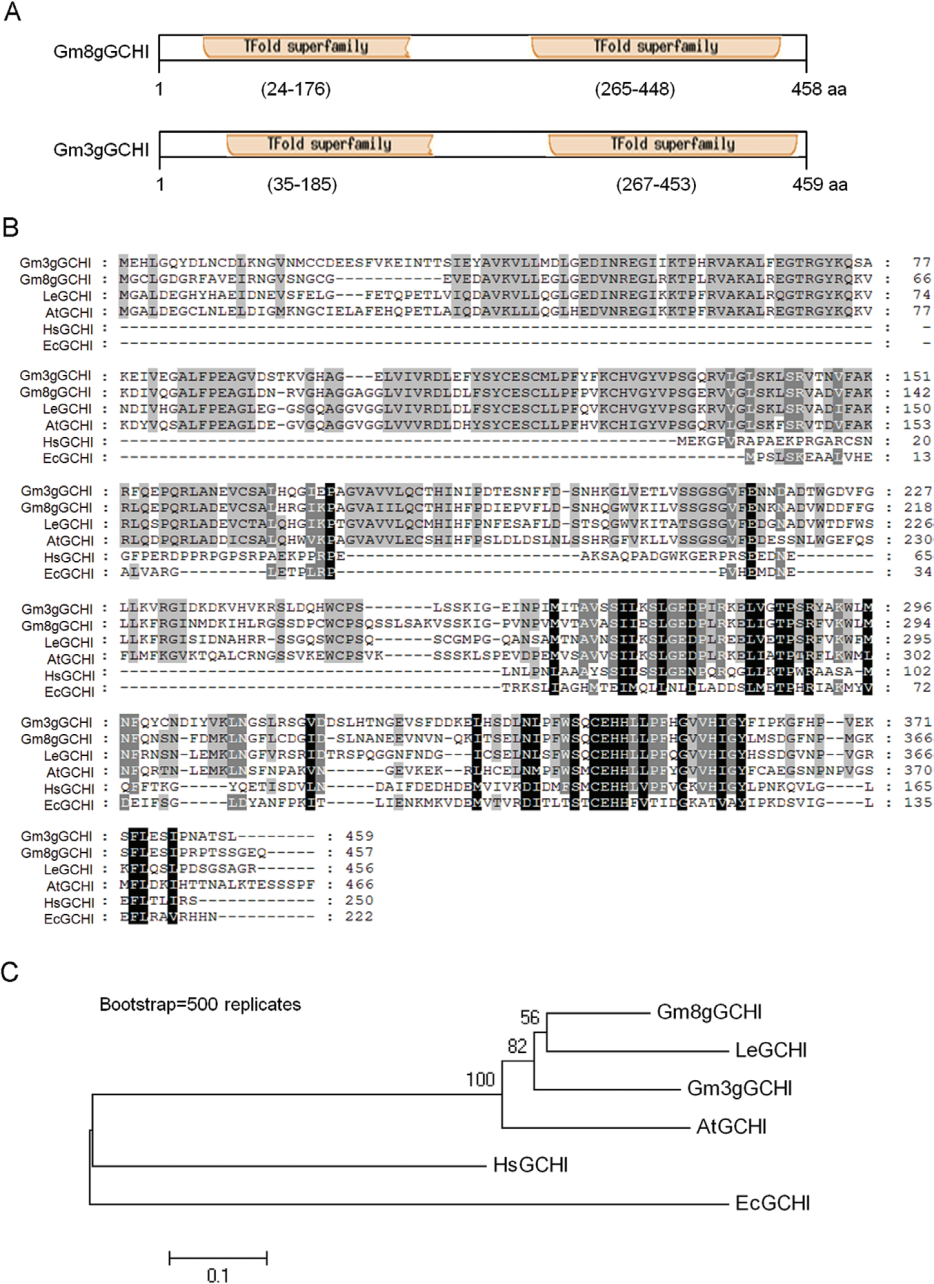
Schematic characterization of GmGCHIs and multiple alignments with other homologs. (A) Conserved domains of the deduced GmGCHI proteins. There are two tunneling fold (TFold) superfamilies in GmGCHIs, localized at ~35–185 aa and ~267–453 aa in Gm3gGCHI, and at ~24–176 aa and ~265–448 aa in Gm8gGCHI. (B) Alignment of GCHI homologs of soybean (Gm), tomato (Le), *Arabidopsis thaliana* (At), *Homo sapiens* (Hs), and *E. coli* (Ec). Identical and similar residues are shaded in black and gray, respectively. Dashes are gaps that maximize the alignment. (C) Phylogenetic analysis of GCHI homologs by use of Neighbor-Joining analysis.

We characterized the *GmGCHI* and *GmADCS* sequences by analysing their predicted proteins. GCHIs have been cloned from *E. coli*, yeast, and mammalian samples, and subsequent crystal structure analysis has shown that *E. coli* and human GCHIs are homodecamers with ~26-kDa subunits, comprised of five tightly associated dimers (Nar *et al.*, 1995; Nardese *et al.*, 1996; Auerbach *et al.*, 2000). In plants, tomato and Arabidopsis GCHIs have been identified as ~50-kDa proteins that contain two GCHI-like domains in tandem. Neither of the domains works in isolation (Basset *et al.*, 2002). Similar to tomato and Arabidopsis GCHIs, Gm3gGCHI was predicted to encode a 459-amino acid (aa) polypeptide and contains two tunneling fold (TFold) superfamily domains, located at ~35–185 aa and ~267–453 aa. Gm8gGCHI encodes 457 amino acids and also contains two TFold superfamily domains at ~24–176 aa and ~265–448 aa (Fig. 2A). The amino-acid sequence of Gm3gGCHI had 67% similarity to that of Gm8gGCHI. The protein domains of the two GmGCHIs are conserved very well across their homologs in tomato (LeGCHI), Arabidopsis (AtGCHI), *Homo sapiens* (HsGCHI), and *E. coli* (EcGCHI) (Fig. 2B). Phylogenetic tree analysis indicated that GmGCHI proteins are closest to the tomato LeGCHI protein (Fig. 2C).

**Fig. 3.**
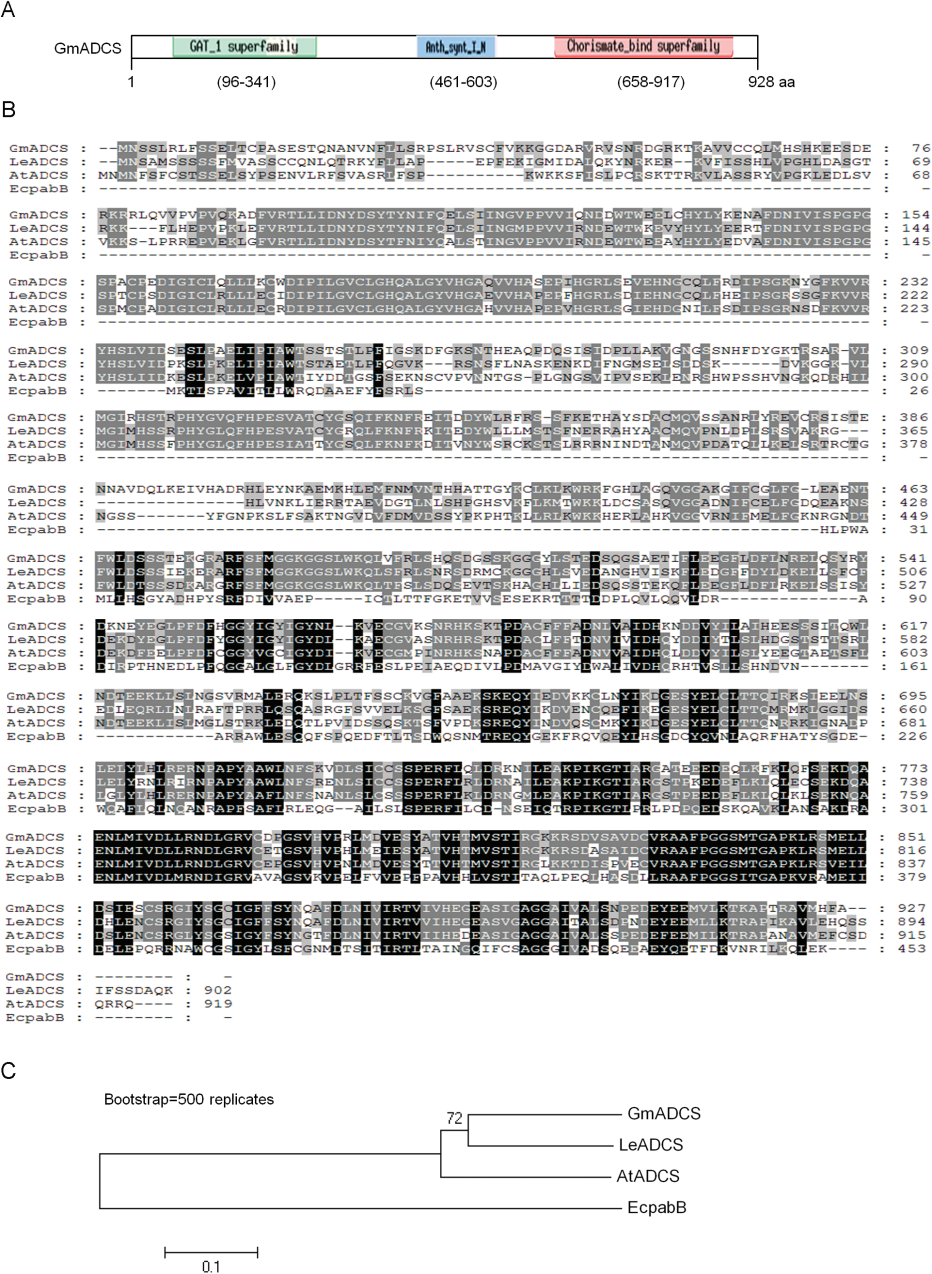
Schematic characterization of GmADCS and multiple alignments with other homologs. (A) Conserved domains of the deduced GmADCS. The GmADCS protein contains three domains including the GAT_1 superfamily (type1 glutamine amidotransferase), Anth_synt_I_N (anthranilate synthase component I, N terminal region), and Chorismate_bind superfamily. (B) Alignment of ADCS homologs of soybean (Gm), tomato (Le), *Arabidopsis thaliana* (At), and *E. coli* (EcpabB). Identical and similar residues are shaded in black and gray, respectively. Dashes are gaps that maximize the alignment. (C) Phylogenetic analysis of ADCS homologs by use of Neighbor-Joining analysis.

The deduced GmADCS protein encoded 927 amino acids and contained three conserved domains, namely a GAT_1 (type 1 glutamine amidotransferase) superfamily, an anthranilate synthase (Anth_synt_I_N) superfamily, and chorismate binding sites, which were located at ~96–341 aa, ~461–603 aa, and ~658–917 aa, respectively, as shown in Fig. 3A. These GmADCS domains were very similar to those of LeADCS, AtADCS, HmADCS, and pabB from EcpabB (*E. coli*) (Fig. 3B). This implies that these enzymes may exhibit conserved functionality. The results of the phylogenetic tree analysis, shown in Fig. 3C, indicated that GmADCS is closest to LeADCS.

### Subcellular localization of GmGCHI-GFP and GmADCS-N-GFP fusion proteins

**Fig. 4.**
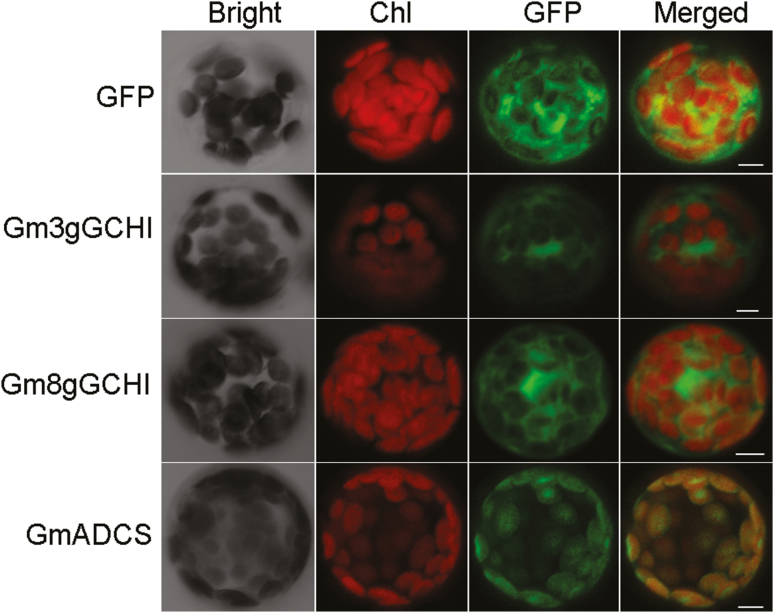
Subcellular localization of GmGCHI-GFP and GmADCS-N-GFP fusion proteins in Arabidopsis mesophyll protoplasts. The coding regions of two *GmGCHI* genes and a 300-bp N-terminal sequence of *GmADCS* were fused with GFP at the C-terminal. The cytoplasm localization of GFP was used as the control; the GFP signal is indicated in green and the chlorophyll autofluorescence (Chl) in red. As the images show, the two GmGCHI fusion proteins were localized to the cytosol with similar signals to GFP. The GmADCS-N terminal fusion protein localized in the chloroplasts as the green signal merges completely with the Chl. The images were scanned using a confocal microscope. The scale bars represent 5 μm.

Given that the folate biosynthesis pathways of plants are compartmented, we analysed the subcellular localization of the three proteins described above by protoplast transient transformation. For this purpose, we fused the full coding sequences of two *GmGCHI* genes and the N-terminal sequence (360 bp) containing the predicted signal peptide sequence of the *GmADCS* gene with GFP protein, respectively, under the control of the *CaMV35S* promoter. The three constructs and the control vector were introduced into protoplasts isolated from Arabidopsis leaves. As shown in Fig. 4, we observed the GFP signals of the two GmGCHI fusion proteins in cytosol and the GFP signal of the GmADCS-N fusion protein in chloroplasts. The subcellular localization of GmGCHI and GmADCS was consistent with the compartmentation of folate biosynthesis, in which GCHI is involved in pterin biosynthesis in the cytosol and ADCS is involved in pABA formation in plastids.

### Effects of *GmGCHI* and *GmADCS* overexpression on levels of folate precursors and folate in Arabidopsis

**Fig. 5.**
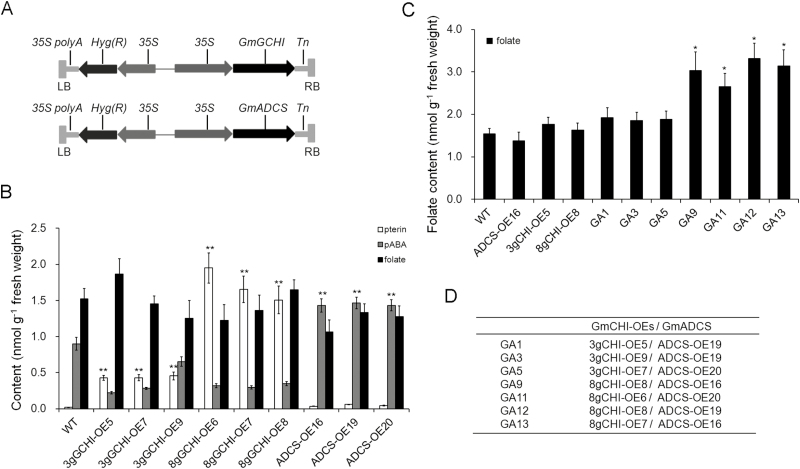
Analysis of folate, pterin, and pABA in transgenic Arabidopsis leaves. (A) Schematic representation of the T-DNA regions of the expression vectors used in Arabidopsis transformation. The coding regions of *GmGCHI* and *GmADCS* were driven by CaMV35S promoters (*35S*). (B) Pterin, pABA, and folate levels in Arabidopsis wild-type (WT), *Gm3gGCHI* overexpression plants (*3gGCHI*-OEs), *Gm8gGCHI* overexpression plants (*8gGCHI-*OEs), and *GmADCS* overexpression plants (*ADCS*-OEs). (C) Folate analysis in crossed plants (GAs) of *GCHI*-OEs/*ADCS*-OEs. (D) The crossing combinations of GA plants. Significant differences compared with the wild-type were determined using Student’s *t*-test: *P<0.05; **P<0.01

To assess the specific roles that the isolated *GmGCHI* and *GmADCS* genes play in folate biosynthesis, we introduced the entire coding regions of *GmGCHI*s and *GmADCS* driven by *CaMV35S* promoters into wild-type Arabidopsis (Fig. 5A). We obtained transgenic plants overexpressing *Gm3gGCHI* (*3gGCHI-OE*), *Gm8gGCHI* (*8gGCHI-OE*), and *GmADCS* (*ADCS-OE*). Pterin contents of GCHI-OE plants were significantly greater than those of the wild-type (Fig. 5B). An average 22-fold increase in pterin content (mainly of hydroxymethylpterin and pterin-6-carboxylic acid) was observed in *3gGCHI-OE* plants and ~79–102-fold in *8gGCHI-OE* plants (*P*<0.01). The average free pABA content of *ADCS-OE* plants overexpressing *GmADCS* was 2-fold greater than that of the wild type (*P*<0.01). However, folate levels of *GCHI-OE* plants did not differ significantly from those of the wild-type and were slightly lower in *ADCS-OE* plants (Fig. 5B). We then crossed the two types of transgenic Arabidopsis plants, thereby obtaining GA (*GCHI-OEs/ADCS-OEs*) plants, to evaluate the effect of the two-gene co-overexpression on folate accumulation. Folate levels of *3gGCHI-OE/GmADCS-OE* cross-plants were ~1.2–1.3 times higher than those of the wild-type. Furthermore, we obtained greater increases in folate (~1.4–1.9 times) in the crosses with *Gm8gGCHI* overexpression (*P*<0.05) (Fig. 5C, D). The results therefore suggest that co-expression of *GmGCHI* and *GmADCS* provides a more effective mechanism for folate accumulation than does the expression of a single gene.

### Co-expression of *GmGCHI* and *GmADCS* in maize and wheat

**Fig. 6.**
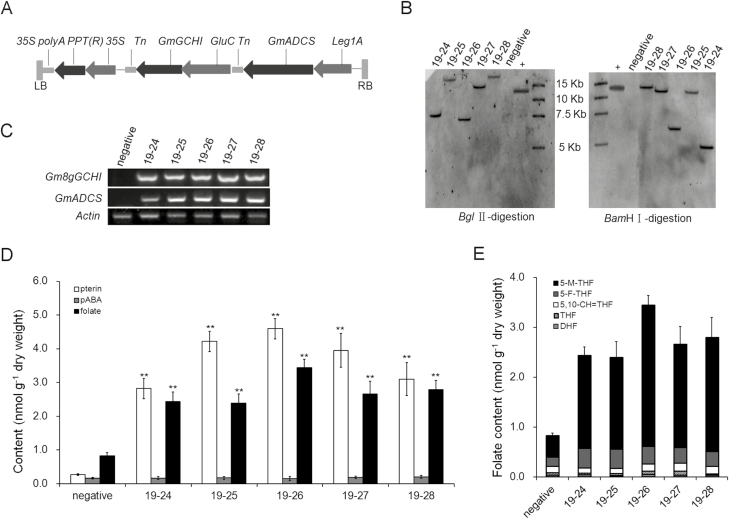
Folate metabolic engineering in maize. (A) Schematic representation of the T-DNA region of the vector used in maize transformation. *GmADCS* and *Gm8gGCHI* were in series under the control of different endosperm-specific *Leg1A* and *GluC* promoters, respectively. (B) The *Bgl* II-digested genomic DNA (left) and the *Bam*H I-digested genomic DNA (right) hybridized with the *GmADCS* probe were both present as a single copy in the transgenic events. + represents the positive control of the plasmid. (C) Expression levels of *Gm8gGCHI* and *GmADCS* in kernels of transgenic maize identified by RT-PCR. (D) Folate, pterin, and pABA levels and (E) the main folate derivatives in grains of transgenic plants in the BC5 generation, and in grains of non-transgenic plants (‘negative’). Significant differences compared with the ‘negative’ control were determined using Student’s *t*-test: **P<0.01

We investigated whether it is potentially feasible to implement folate biofortification by simultaneously engineering the pterin and pABA branches in maize and wheat. The sequences encoding the *Gm8gGCHI* and *GmADCS* genes were driven by endosperm-specific promoters from the rice *GluC* gene and maize *Leg1A* gene, respectively, and were linked to the expression vector *pCAMBIA3301* in a single T-DNA region (Fig. 6A). We applied an *Agrobacterium*-mediated transformation to introduce the *Gm8gGCHI*-*GmADCS*-*pCAMBIA3301* vector into the maize cultivar HiII. In addition, the T-DNA expression cassette was introduced into the maize inbred line Zheng58 by backcrossing, until the BC5 generation was obtained. We confirmed that the exogenous folate genes were integrated into the genome as a single copy by Southern blotting (Fig. 6B) and the introduced genes were abundantly expressed by RT-PCR (Fig. 6C). We evaluated the folate concentrations of mature grains in transgenic maize, and used grains from non-transgenic plants segregated from the heterozygous transgenic plants as controls. The average folate content of transgenic plants was ~3–4.2 times greater than that of the control plants (Fig. 6D). For example, the folate levels were significantly enhanced in transgenic maize 19–26 in comparison with the control (3.43±0.25 versus 0.82±0.09 nmol g^–1^ dry weight; *P*<0.01). Meanwhile, we detected very high pterin contents, mainly of hydroxymethylpterin and pterin-6-carboxylic acid, in transgenic maize. The pterin contents were up to ~10–17 times higher than those of the control (*P*<0.01). Unexpectedly, there were no large differences in the pABA content between the control and transgenic maize (Fig. 6D). 5-Methyltetrahydrofolate (5-M-THF) and 5-formyltetrahydrofolate (5-F-THF) are the two major folate derivatives found in maize grains. The 5-M-THF and 5-F-THF levels were significantly higher in transgenic grains than in the control grains (Fig. 6E). Specifically, 5-M-THF was elevated most, accounting for up to 82% of the total folate content in transgenic maize 19–26.

**Fig. 7.**
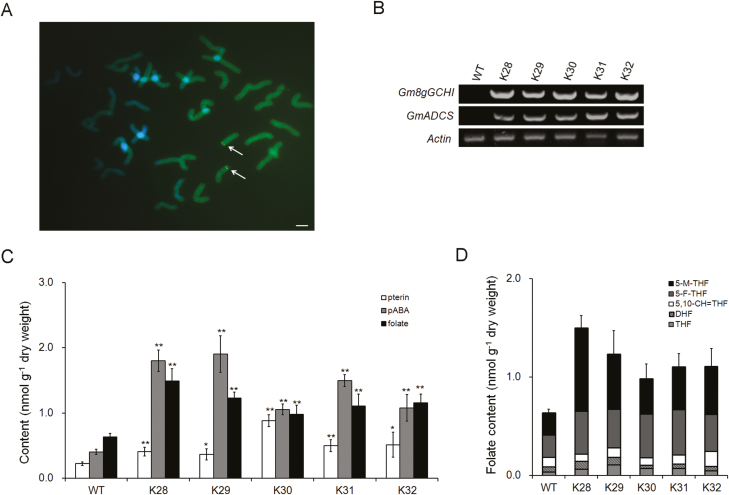
Folate metabolic engineering in wheat by co-expression of *GmGCHI* and *GmADCS*. (A) Fluorescence *in situ* hybridization analysis of the introduced T-DNA cassette in the homozygous transgenic wheat plants. Arrows indicate positive blots. The scale bar is 5 μm. (B) Expression of *Gm8gGCHI* and *GmADCS* in the kernels of transgenic wheat plants compared with the wild-type (WT). (C) Folate, pterin, and pABA levels and (E) the main folate derivatives in mature grains of the wild-type (WT) and transgenic wheat. Significant differences compared with the wild-type were determined using Student’s *t*-test: *P<0.05; **P<0.01.

We then introduced the *Gm8gGCHI-GmADCS-pCAMBIA3301* co-expression vector into the wheat cultivar Fielder by *Agrobacterium*-mediated transformation and homozygous transgenic plants were obtained using the haploid-doubling technique. We confirmed the insertion of the T-DNA cassette by fluorescence *in situ* hybridization in homozygous plants (Fig. 7A). RT-PCR detected high levels of *Gm8gGCHI* and *GmADCS* expression in the developing grains of the transgenic wheat (Fig. 7B). Folate analysis of the mature grains revealed that the average folate contents of homozygous transgenic wheat were 2.3 times higher than those of the wild-type (1.49±0.11 versus 0.63±0.05 nmol g^–1^ dry weight, ~65 μg/100 g versus ~28 μg/100 g; *P*<0.01) (Fig. 7C). Moreover, we detected a ~2.6–4.7-fold increase in the free pABA levels and a ~1.8–4.0-fold increase in the pterin levels (mainly of hydroxymethylpterin and pterin-6-carboxylic acid) in transgenic grains (*P*<0.01) (Fig. 7C). 5-M-THF and 5-F-THF were the major folate derivatives in wheat grains. For example, 5-M-THF and 5-F-THF constituted 46% and 16%, respectively, of the total folate in transgenic wheat K28 (Fig. 7D).

### Co-expression of codon-optimized *GmGCHI* and tomato *ADCS* in wheat

**Fig. 8.**
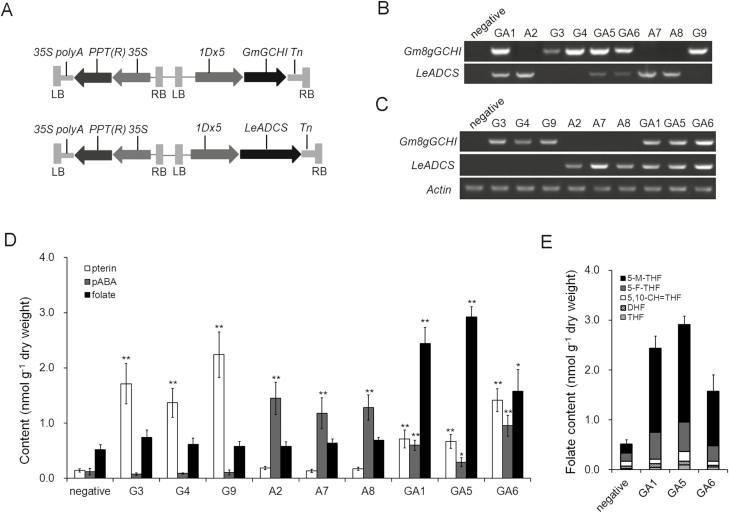
Folate engineering in wheat by optimized *GmGCHI* and *LeADCS*. (A) Schematic representation of the T-DNA regions of the vector used in wheat transformation. (B) Identification of three types of transgenic wheat by PCR amplification of the target genes using genomic DNA as templates: *Gm8gGCHI*^*+*^ (G), *LeADCS*^*+*^(A), and *GmGCHI*^*+*^*/LeADCS*^*+*^ (GA). Non-transgenic wheat is indicated as ‘negative’. (C) expression levels of *Gm8gGCHI* and *GmADCS* in transgenic wheat kernels. (D) Folate, pterin, and pABA levels and (E) the main folate derivatives in transgenic wheat grains and grains of the negative wheat without the target genes. Significant differences compared with the ‘negative’ control were determined using Student’s *t*-test: *P<0.05; **P<0.01

We investigated the possibility of further enhancing the folate content of wheat grains by use of the codon-optimized tomato gene *LeADCS* and soybean gene *Gm8gGCHI*. The two target genes, controlled by a wheat endosperm-specific glutenin (*1Dx5*) promoter, were constructed into the vector *pWMB122*, which contained double T-DNAs to obtain marker-free transgenic plants (Fig. 8A). Then, we co-introduced the two vectors *Gm8gGCHI-pWMB122* and *LeADCS*-*pWMB122* to the wheat cultivar Fielder by *Agrobacterium*-mediated transformation. PCR detection of the target genes identified three types of transgenic plants carrying either *Gm8gGCHI*^*+*^ (G) or *LeADCS*^*+*^ (A), or both *Gm8gGCHI*^*+*^ and *LeADCS*^*+*^ (GA) (Fig. 8B), and RT-PCR analysis revealed high expression of *Gm8gGCHI* and *LeADCS* in the transgenic wheat grains (Fig. 8C).

We assessed the levels of folates and their precursors in the mature grains of the transgenic wheat plants, again using the non-transgenic plants segregated from heterozygous transgenic plants as controls. The levels of the folate precursor pterin were up to 16 times higher in the G grains than that of the control (*P*<0.01); however, no significant changes were observed in pABA levels and folates were increased only slightly (1.4-fold). pABA was increased by up to 12-fold in the A grains (*P*<0.01); however, no significant changes in pterin levels were detected, and only a slight increase (1.3-fold) in folate content was observed, compared to the control. In GA plants containing both the target genes (*Gm8gGCHI*^*+*^/*LeADCS*^*+*^), folate levels increased by up to 5.6-fold (2.91±0.19 versus 0.52±0.05 nmol g^–1^ dry weight for GA and controls grains, respectively; *P*<0.01) (Fig. 8D). In addition, the concentrations of pterin (mainly hydroxymethylpterin and pterin-6-carboxylic acid) and free pABA in the GA grains increased by ~4.7–10-fold and ~2.4–8 fold, respectively, compared to those of the control. 5-M-THF and 5-F-THF, the two major folate species, accounted for 67% and 20% of the total folates in the transgenic grains, respectively, and were significantly increased in the transgenic wheat GA grains (Fig. 8E).

## Discussion

Enhancing folate accumulation in food crops by applying metabolic engineering is regarded as an important strategy with great promise for alleviating folate malnutrition (Blancquaert *et al.*, 2014). GTP cyclohydrolase I (GCHI) and aminodeoxychorismate synthase (ADCS) are two key enzymes that catalyse the formation of the folate precursors pterin and p-aminobenzoate, respectively, and the reactions that they perform are also considered as rate-limiting steps in folate biosynthesis (Díaz de la Garza *et al.*, 2007; Storozhenko *et al.*, 2007). In this study, biofortified maize and wheat with improved folate levels were created by co-overexpression of these two genes. First, we successfully cloned the soybean *GmGCHI* and *GmADCS* genes by combining EST-based amplification and the RACE technique (Supplementary Figs S1, S2). We then conducted a transgenic analysis of the two soybean genes in Arabidopsis, which confirmed that their roles in the biosynthesis of the folate precursors pterin and pABA were conserved. This provides the first report of the cloning and characterization of genes involved in soybean folate biosynthesis. The genes were then used in metabolic engineering.

Folate enhancement in transgenic Arabidopsis plants was not observed with the single genes *GmGCHI*^*+*^ or *GmADCS*^*+*^; however, a ~1.2–1.9-fold increase was achieved when both genes were carried (Fig. 5). Similarly, the folate levels of transgenic wheat grains expressing the single genes *GmGCHI*^*+*^ or *LeADCS*^*+*^ were only increased by 1.4-fold, whereas levels in *GmGCHI*^*+*^*/LeADCS*^*+*^ wheat grains were increased by 5.6-fold (Fig. 8). We therefore conclude that the two-gene strategy of simultaneously boosting both pterin and pABA has greater potential for enhancing folate accumulation in Arabidopsis and wheat than a single-gene strategy.

Folate biofortification via overexpression of the *GCHI* and *ADCS* genes has been very successful in tomato (25-fold) and rice (100-fold) (Díaz de la Garza *et al.*, 2007; Storozhenko *et al.*, 2007), and has had modest success in potato (3-fold) (Blancquaert *et al.*, 2013). In our study, we increased the folate contents of maize and wheat by ~2–6-fold. Taking all these findings together, we presume that regulation of folate biosynthesis varies among plant species. Other possible limiting factors are predicted to affect the potential of folate biofortification in plants. In *GCHI*^*+*^/*ADCS*^*+*^ potatoes, introduction of the genes *HPPK/DHPS* and *FPGS* augmented folate levels up to 9-fold (De Lepeleire *et al.*, 2018); thus, the bottlenecks were predicted to arise from other folate biosynthesis genes. Similar to *GCHI*^*+*^/*ADCS*^*+*^ potatoes (Blancquaert *et al.*, 2013), we also observed considerable increases in the folate precursors pterin and pABA in transgenic wheat grains with *GmGCHI*^*+*^*/LeADCS*^*+*^ (Fig. 8C) but only modest increases in folate levels. Therefore, there may be other limiting factors that affect folate biosynthesis in wheat. In the case of maize, the transgene expression increased folates and pterin by ~3–4.2 fold and ~10–17 fold, respectively, but it had no impact on pABA levels (Fig. 6D). This observation led us to conclude that the pABA branch might be more tightly regulated than the pterin branch in maize, thus resulting in the modest enhancement of folates. In contrast, an increase in the pterin branch has been shown to cause a significant rise in pABA levels in genetically engineered common bean (Ramírez Rivera *et al.*, 2016). Thus, it seems that endogenous regulation of folate biosynthesis in plants is somewhat complicated and a case-by-case approach should be taken when folate biofortification is pursued via metabolic engineering.

The biosafety of genetically modified crops remains an issue of great public concern. It would be helpful to remove selectable markers from transgenic plants, as this would alleviate public concerns regarding genetically engineered crop varieties escaping into local ecosystems (Wang *et al.*, 2018). Several strategies can be used to generate marker-free transgenic plants (Li *et al.*, 2012; Tuteja *et al.*, 2012; Wang *et al.*, 2017), and double T-DNA-mediated co-transformation has been successfully applied for this purpose in many species (Xing *et al.*, 2000; Miller *et al.*, 2002; Lu *et al.*, 2009; Ramana Rao *et al.*, 2011; Wang *et al.*, 2017). In our study, transgenic wheat plants with significant improvement of folate accumulation were generated by co-transformation of folate genes with the selectable marker gene *bar* (Fig. 8). In future studies, we will focus on transgenic events that do not carry *bar* gene.

## Supplementary data

Supplementary data are available at *JXB* online.

Fig. S1. Blast of soybean ESTs with the *AtGCHI* and *AtADCS* coding sequences and primer maps for cloning and identification of *GmGCHI*s and *GmADCS*.

Fig. S2. Amplification of *GmGCHI* and *GmADCS*.

Table S1. Primers used in cloning and identification of *GmGCHI* genes.

Table S2. Primers used in cloning and identification of *GmADCS* and *LeADCS*.

Supplementary Figures S1-S2 and Tables S1-S2
