# Supplementary material for: Improved folate accumulation in genetically modified maize and wheat
Source: J Exp Bot. 2019 Feb 8;70(5):1539–51. doi: 10.1093/jxb/ery453 (PMC6411382; doi:10.1093/jxb/ery453)
Supplement: Supplementary Figures S1-S2 and Tables S1-S2 [file ery453_suppl_supplementary_figures_s1-s2_tables-s1-s2.pdf]

Supplementary data

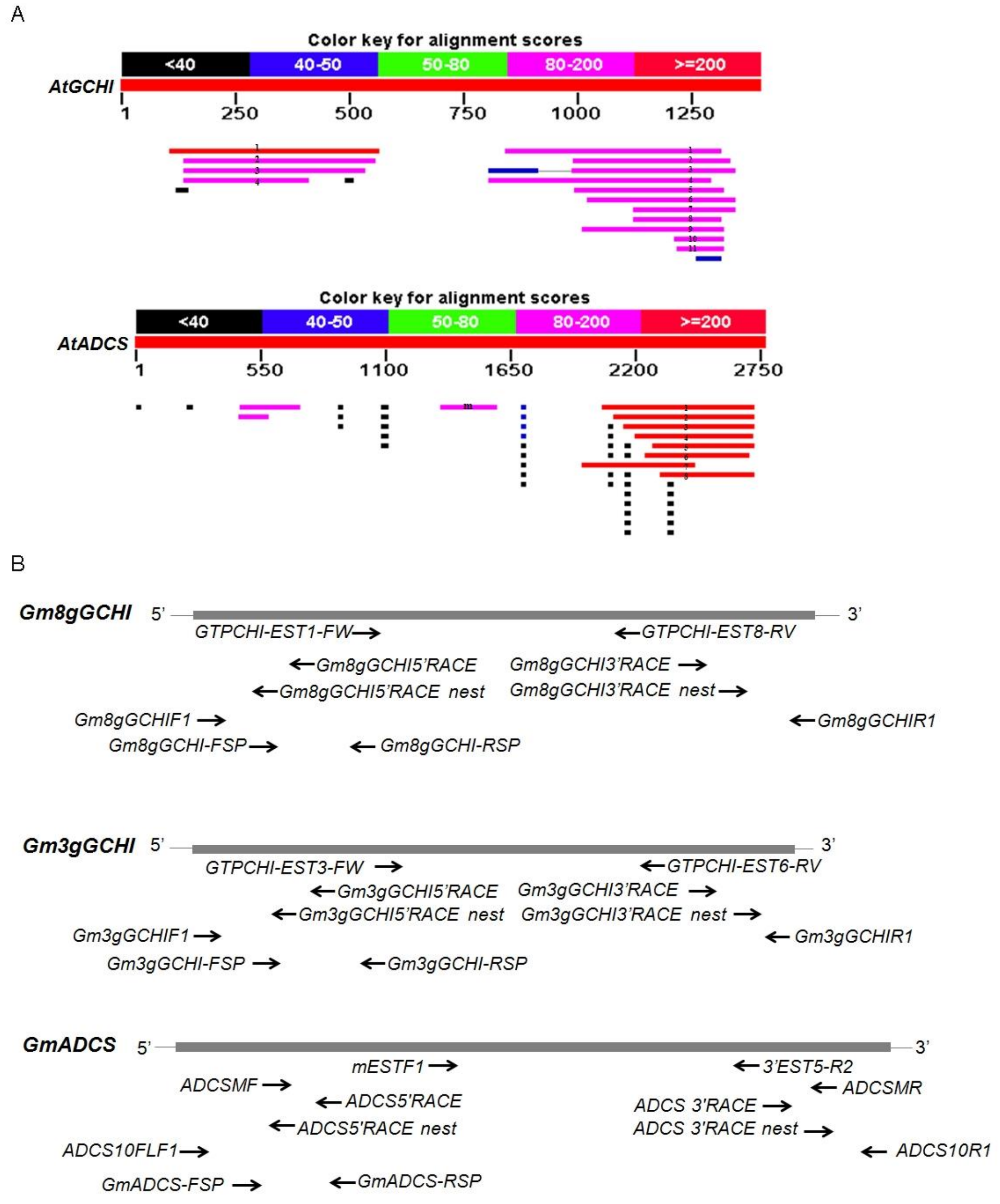

Figure S1. Blast of soybean ESTs with *AtGCHI* and *AtADCS* coding sequences (A) and primer maps for cloning and identification of *GmGCHIs* and *GmADCS* (B). A, in the soybean EST database, four ESTs similar to the *AtGCHI* 5' terminal sequence and eleven ESTs similar to the 3' terminal sequence were found. Two ESTs similar to the 5' terminal sequence of *AtADCS*, one EST similar to the *AtADCS* middle sequence, and eight ESTs similar to the 3' terminal sequence were observed. Alignment score represents the lengths of ESTs with high similarity to the query sequences of *AtGCHI* and *AtADCS*, and they are showed with different colors: lower than 40 base pairs with black color, 40-50 base pairs with blue color, 50-80 base pairs with green, 80-200 base pairs with pink and more than 200 base pairs with red color. The numbers below AtGCHI (1, 250, 500, 750, 1000, 1250) and AtADCS (1, 550, 1100, 1650, 2200, 2750) represent the length scale. EST, expressed sequence tag. B, sketch maps of primers used for cloning and identification of *Gm8gGCHI*, *Gm3gGCHI* and *GmADCS*.

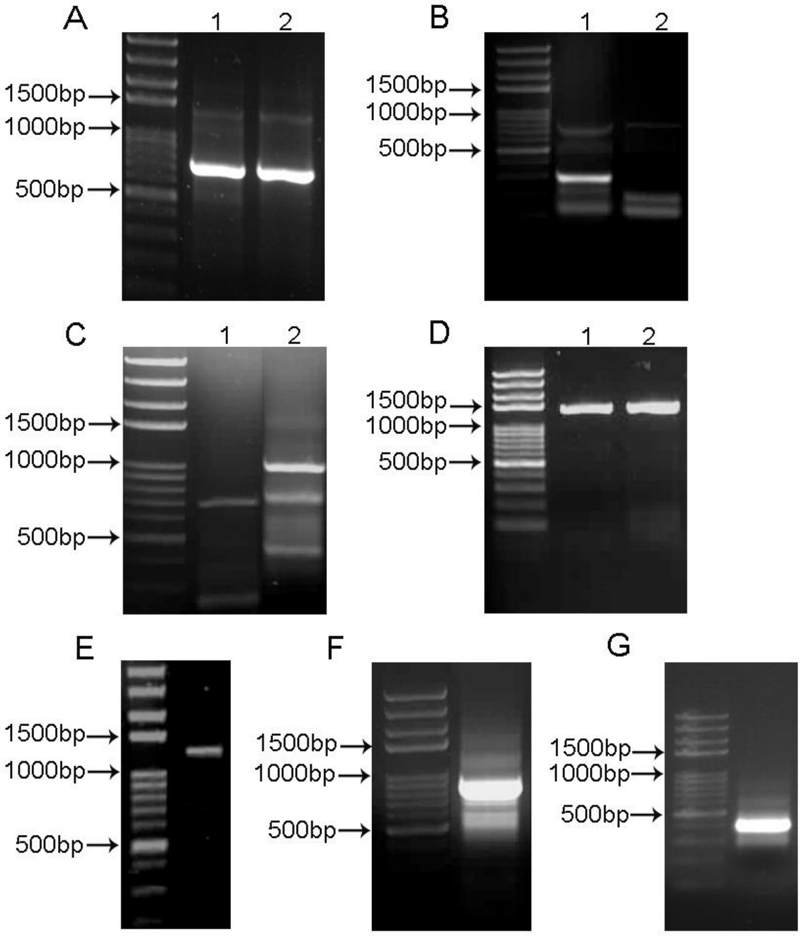

Figure S2. Amplification of *GmGCHI* and *GmADCS*. A, partial PCR products of *GmGCHI* genesobtained by primers: 1, *GTPCHI-EST1-FW* + *GTPCHI-EST8-RV*; 2, *GTPCHI-EST3-FW* + *GTPCHI-EST6-RV*. B, 5’ RACE and 3’ RACE products of *Gm3gGCHI*; 1, a 300-bp bright band obtained by 5’RACE; 2, small and non-specific bands obtained by 3’RACE. C, 5’ RACE and 3’ RACE products of *Gm8gGCHI*; 1, a 700-bp product obtained by 5’ RACE; 2, products obtained by 3’ RACE. D, amplification of the full coding regions of *GmGCHIs*; 1, *Gm3gGCHI*; 2, *Gm8gGCHI*. E, a 1300-bp 3’terminal product of *GmADCS* amplified by use of primers *mESTF1* + *3’EST5-R2*. F, a 800-bp product of *GmADCS* obtained by 5’ RACE. G, a 300-bp product of *GmADCS* obtained by 3’RACE. We used a 100-bp plus marker for each pattern. The complete bands were including 100, 200, 300, 400, 500, 600, 700, 800, 900, 1000, 1500, 2000, 3000 and 5000 bp from down to up, and 500 bp, 1000 bp and 1500 bp bands in A-G were marked with arrows.

Table S1. Primers used in cloning and identification of *GmGCHI* genes.

Primer names	Sequence 5’-3’
GTPGCHI-EST1-FW	TCAAGTGTCATGTGGGTTATGTC
GTPGCHI-EST3-FW	CATGTGGGTTATGTCCCTTCTG
GTPGCHI-EST-6RV	TGCACTATTGACTGTAAAAGG
GTPGCHI-EST-8RV	CCTGAACTTGGAGCTTGAAAC
Gm3gGCHI5’RACE	CCTTCGCACTTTGCTTGTAACCTC
Gm3gGCHI5’RACE nest	GTGGTGTCTTTATAATGCCTTCCCTG
Gm8gGCHI5’RACE	CATCTTCCCCTAGACCCTCCAAC
Gm8gGCHI5’RACE nest	CAGCATCCTCAACTTCACCACAAC
Gm3gGCHI3’RACE	GTGATAGTGGTGGTGGAAGCAAGTC
Gm3gGCHI3’RACE nest	GACCTTGCTGCAAGAACCTCGTTTC
Gm8gGCHI3’RACE	GTAGAGGCAAGCCACACATGTATG
Gm8gGCHI3’RACE nest	CGAGGGGAATTGAGAAGTTTGGAAG
Gm3gGCHI-FSP	AGTCTTTTGTCAAAGAGATCAATACAACTT
Gm3gGCHI-RSP	GAATCGTTTTGCAAACACATTGGTC
Gm8gGCHI-FSP	GATGGGCGTTTCGCTGTTGAGAT
Gm8gGCHI-RSP	GAAGTCGTTTCGCAAATACATCAGCA
Gm3gGCHIF1	ATGGAGCATTTGGGTCAGTATG
Gm3gGCHIR1	TCAAAGAGATGTAGCATTTGG
Gm8gGCHIF1	ATGGGGTGTTTGGGTGATGGGCG
Gm8gGCHPF1	ATGGGGTGTCTGGGGGATGGGCG
Gm8gGCHPR1	CTACTGCTCCCCAGATGATGTAG
Gm8gGCHI-PFSP	GAGCAGGTGGACTCGTGATTG
Gm8gGCHI-PRSP	GCAGATAGAGAAGACTGAGAAG

Table S2. Primers used in cloning and identification of *GmADCS* and *LeADCS*.

Primer names	Sequence 5’-3’
3’EST5-R2	CTATTGTTTGGCTTATGAGCTC
mESTF1	GGATCACTTTGGAAGCAGTTAG
ADCSMF	GCATTTGTCTTCAGCTGTTG
ADCSMR	CTAAGCAAAATGCATCACAG
ADCS5'RACE	CCTCTGTGTCATTCAACCACTGTG
ADCS5'RACE nest	GACGGTTCGATTTCACACCACACTC
ADCS 3'RACE	CCTGGTGGTTCAATGACAGGTGCAC
ADCS 3'RACE nest	CAGTCATTGTACACGAGGGTGAAG
ADCS10FLF1<br>*ADCS10R1*	ATGAATTCGTCTCTGCGTTTG<br>CTAAGCAAAATGCATCACAGC
LeADCSPF1	ATGAATAGCGCAATGTCCTCCTC
LeADCSPR1	TCACTTTTGTGCGTCGCTGCTG
GmADCS-FSP	GATGATTGGACATGGGAAGAAC
GmADCS-RSP	GATCTGGCTGAGCTTCATGAG
LeADCS-FSP	ACGGGAGGCTTAGCGACATTG
LeADCS-RSP	TCTTTGAGCGGTGCCTGTTAG
TaActinF	CTCACTGAGGCTCCTTTGAAC
TaActinR	CGAGATCCAAACGAAGAATG
ZmActinF	ATGTTTCCTGGGATTGCCGAT
ZmActinR	CCAGTTTCGTCATACTCTCCCTTG
